# Prognostic Nomogram for Thoracic Esophageal Squamous Cell Carcinoma after Radical Esophagectomy

**DOI:** 10.1371/journal.pone.0124437

**Published:** 2015-04-20

**Authors:** Dan Su, Xinming Zhou, Qixun Chen, Youhua Jiang, Xun Yang, Weihui Zheng, Kaiyi Tao, Jie Wu, Zhen Yan, Liang Liu, Shaoyuan Wu, Weimin Mao

**Affiliations:** 1 Cancer Research Institute, Zhejiang Cancer Hospital & Key Laboratory Diagnosis and Treatment Technology on Thoracic Oncology of Zhejiang Province, Hangzhou, China; 2 Department of Thoracic Surgery, Zhejiang Cancer Hospital, Hangzhou, China; 3 Department of Statistics and Institute of Bioinformatics, University of Georgia, Athens, Georgia, United States of America; 4 Department of Biochemistry and Molecular Biology, and Tianjin Key Laboratory of Medical Epigenetics, School of Basic Medical Sciences, Tianjin Medical University, Tianjin, China; Chinese Academy of Medical Sciences, CHINA

## Abstract

Nomogram has demonstrated its capability in individualized estimates of survival in diverse cancers. Here we retrospectively investigated 1195 patients with esophageal squamous-cell carcinoma (ESCC) who underwent radical esophagectomy at Zhejiang Cancer Hospital in Hangzhou, China. We randomly assigned two-thirds of the patients to a training cohort (n = 797) and one-third to a validation cohort (n = 398). Cox proportional hazards regression analyses were performed using the training cohort, and a nomogram was developed for predicting 3-year and 5-year overall survival rates. Multivariate analysis identified tumor length, surgical approach, number of examined lymph node, number of positive lymph node, extent of positive lymph node, grade, and depth of invasion as independent risk factors for survival. The discriminative ability of the nomogram was externally determined using the validation cohort, showing that the nomogram exhibited a sufficient level of discrimination according to the C-index (0.715, 95% CI 0.671–0.759). The C-index of the nomogram was significantly higher than that of the sixth edition (0.664, *P*-value<0.0001) and the seventh edition (0.696, *P*-value<0.0003) of the TNM classification. This study developed the first nomogram for ESCC, which can be applied in daily clinical practice for individualized survival prediction.

## Introduction

China has the highest incidence of esophageal cancer of the world, which represents the fifth most prevalent cancer and the fourth leading cause of cancer-related deaths in China[1–3]. In 2008, it was estimated that there were 259,235 newly diagnosed cases and 211,084 deaths from esophageal cancer in China, accounting for more than half of all the new cases and deaths of this cancer worldwide[4]. Esophageal squamous cell carcinoma (ESCC), the predominant histologic type of esophageal cancer in China, has underwent a steady decrease of incidence in the United States and other countries over the past several decades, but is still increasing in some areas of China[5–8].

Surgery has been considered as the mainstay treatment for patients diagnosed with resectable ESCC[9,10]. Despite recent improvement of surgical techniques, however, the overall survival of patients treated with surgery is still poor because of the loco-regional recurrence and distant metastasis[11,12]. To improve the outcome of ESCC, multimodal therapy that combines chemotherapy and/or radiotherapy with radical surgery has been developed[10,13]. Recently, neoadjuvant chemoradiotherapy followed by esophagectomy has been introduced as an acceptable therapy approach[14–16]. However, the benefits of adjuvant therapy after surgery in long-term survival of ESCC patients remain controversial[17–21]. According to the National Comprehensive Cancer Network (NCCN) Guidelines Version 2.2013, ESCC patients after R0 resection are recommended to observe, regardless whether lymph nodes are positive or negative. On the other hand, recent studies have suggested that postoperative adjuvant therapy may be able to improve survival of ESCC patients with certain clinicopathological features[17,21–23]. An accurate estimation of the survival rates of ESCC patients treated purely with radical esophagectomy is therefore needed in order to determine whether adjuvant therapy would be a useful addition to the standard course of surgical treatments for this disease.

The tumor node metastasis (TNM) classification of the American Joint Committee on Cancer (AJCC) is the most widely used staging system for clinical treatments and prognostic estimates in patients with cancer, including ESCC[24]. The seventh edition of TNM classification stratifies esophageal cancer into seven stages, based on pathological assessment of five risk factors, including depth of tumor invasion (T), number of positive lymph nodes (N), presence of distant metastases (M), tumor location, and grade[25]. However, other clinicopathological factors, such as age, tumor length, number of examined lymph nodes, which may also influence patient outcome, were not considered by the TNM system[26–29].

Nomogram has been widely and successfully used for predicting survival with diverse cancers. Compared to the AJCC TNM staging system, nomogram has the advantages to quantify risk by taking all known clinicopathological variables into account, allowing individualized prognostic prediction in the majority of cancer types[30–37]. To date, however, no nomogram has been developed for ESCC, and the few nomograms reported were derived from western populations for esophageal adenocarcinoma[38,39]. The present study, therefore, represents the first effort to develop a prognostic nomogram for ESCC based on a large cohort of patients who underwent esophagectomy. In addition, this study compared the predictive accuracy of the nomogram to that of both the sixth and seventh AJCC TNM classifications, showing that our nomogram outperformed the TNM staging system for prognostic prediction of survival of ESCC patients.

## Methods

### Ethics statement

The study proposal has been reviewed and approved by Human subject Research Ethics committee of Zhejiang Cancer Hospital and found to conform to the guidelines set forth by this committee. The study did not constitute harm and potential risks to donors. All samples were obtained with written informed consent from all participants.

### Data set

Between January 1 of 2000 and December 31 of 2009, we collected data for 2016 patients who underwent esophagectomy for ESCC at the Zhejiang Cancer Hospital of Hangzhou, China. Surgeries were performed by experienced surgeons of the Zhejiang Cancer Hospital. A total of 1195 eligible patients were enrolled in this study according to the following criteria: primary resectable thoracic esophageal cancer, no combined malignancy, squamous cell carcinoma, no neoadjuvant radiotherapy or chemotherapy and no postoperative radiotherapy or chemotherapy, radical surgery for esophageal carcinoma, R0 resection, and no missing data.

Clinicopathological factors of the data set include tumor location, tumor length, surgical approach, number of examined lymph nodes, number of positive lymph nodes, extent of positive lymph nodes, grade, depth of invasion, and vessel infiltration. Demographic characteristics of patients include age, sex, body mass index (BMI), smoking history, and alcohol intake. The follow-up data includes the follow-up duration and survival status. Because our data were collected prior to the widespread acceptance of Circumferential Resection Margin (CRM) as an important pathological factor, it is unknown which patients in our study were CRM positive.

Tumor location was categorized as upper, middle and lower. Surgical approach was recorded as Ivor Lewis esophagectomy, McKeown esophagectomy and left transthoracic esophagectomy. Extent of positive lymph nodes was categorized as cervical part, upper mediastinum, lower mediastinum, and abdomen. Grade was recorded as well-differentiated, moderately differentiated, poorly differentiated, and undifferentiated. TNM stages of patients were determined according to the sixth and seventh AJCC TNM classifications, respectively[24,40].

Continuous variables were binned into discrete categories according to clinical reasoning or statistical methods in this study. Age was grouped as ≤ 50 years, 51–60 years, 61–70 years, and ≥71 years. BMI was grouped as <18.5 kg/m^2^, 18.5–24.49 kg/m^2^, and ≥24.5 kg/m^2^. Tumor length was grouped as <3 cm, 3–4.9 cm, 5–7.9 cm, and ≥8cm. Number of examined lymph nodes was grouped as <10, 10–19, 20–29, 30–39, 40–49, and ≥50. Number of positive lymph nodes was grouped as 0 (N0), 1–2 (N1), 3–6 (N2) and ≥7 (N3), and depth of invasion was categorized as epithelial lamina (T0), lamina propria or muscularis mucosae (T1a), submucosa (T1b), muscular layer (T2), adventitia (T3) and adjacent structure (T4), according to seventh edition of AJCC TNM staging.

Follow-up was conducted every three months for the first three years, every six months for the next two years, and once a year after five years. Follow-up of the study was complete in the May of 2011, and achieved a minimal follow-up duration of 18 months in the data set. Overall survival (OS) was measured from the time of surgery to the last date of follow-up. The survival status of patients was collected at the last date of follow-up.

### Statistical Analysis

We calculated the Kaplan-Meier estimate of the overall survival curve. The survival curves for different variable groups were compared by the log-rank test. For the development of nomogram, we randomly divided two-thirds of the whole data into a training cohort (n = 797) and one-third into an external validation cohort (n = 398). Data sets were summarized using standard descriptive statistics and frequency tabulation.

Multivariate analyses were performed using Cox proportional hazards regression. Variables were tested for the proportional hazards (PH) assumption based on the scaled Schoenfeld residuals, and variables that violate the PH assumption were removed from the study. Significant explanatory variables for predicting survival times were identified in the training cohort using a backward step-down selection method with Akaike information criterion (AIC). We constructed a nomogram for predicting 3-year and 5-year overall survival probabilities, based on the predictive model with significant explanatory variables.

The utility of the nomogram was evaluated by both internal and external methods. The training cohort was internally validated with bootstrap resampling. External validation was assessed using the validation cohort. Both validations were assessed with respect to discrimination and calibration. Discrimination, which assesses the ability of the model to separate subject outcome, was determined by using the concordance index (Harrell’s C-index)[41,42]. Calibration was performed by plotting the means of Kaplan-Meier estimates with the nomogram predicted survival probabilities. We used the bootstrap technique with 200 repetitions to evaluate the uncertainty in both discrimination and calibration analyses.

Comparisons between the nomogram and the AJCC TNM stage systems were performed using the rcorrp.cens function in the R package Hmisc. All statistical analyses were performed using SPSS 13.0 for Windows (Chicago, IL), and R software version 2.15 (http://www.r-project.org) with the rms, Hmisc, peperr and MASS packages. *P*-value <0.05 in a two-tailed test is considered statistically significant.

## Results

The clinicopathological characteristics of the training cohort (n = 797) and validation cohort (n = 398) are provided in Table 1. There were 510 (64%) patients in the training cohort, and 253 (63.6%) patients in the validation cohort with a history of alcohol intake. The mean numbers of examined lymph nodes were 24.8 and 24.6 in the training and validation cohorts, respectively. There were 64.5% and 67.8% patients respectively in the training and validation cohorts who were removed more than 20 lymph nodes.

**Table 1:** Patient clinicopathologic characteristics of the training and validation cohorts.

Variable	Training set (n = 797)		Validation set (n = 398)	
	No. of Patients	%	No. of Patients	%
**Sex**				
Male	688	86.3	345	86.7
Female	109	13.7	53	13.3
**Age (years)**				
≤50	132	16.6	73	18.3
51–60	317	39.8	154	38.2
61–70	272	34.1	136	34.2
≥71	76	9.5	35	8.8
Mean, Median (Range)	59.03,59.0 (34–80)		58.85,59 (37–97)	
**Body Mass Index (kg/m** ^**2**^ **)**				
<18.5	111	13.9	37	9.3
18.50–24.49	606	76.0	321	80.7
≥24.50	80	10.0	40	10.1
Mean, Median (Range)	21.3,21.1 (14–30)		21.4,21.2 (15–31)	
**Smoking history**				
No	209	26.2	114	28.6
Yes	588	73.8	284	71.4
**Alcohol intake**				
No	287	36.0	145	36.4
Yes	510	64.0	253	63.6
**Tumor location**				
Upper	21	2.6	7	1.8
Middle	392	49.2	197	49.5
Lower	384	48.2	194	48.7
**Tumor length (cm)**				
<3.0	137	17.2	67	16.8
3.0–4.9	325	40.8	162	40.7
5.0–7.9	292	36.6	148	37.2
≥8	43	5.4	21	5.3
Mean, Median (Range)	4.41,4.0 (0–14)		4.42,4.05 (0–10)	
**Grade**				
Well-differentiated	125	15.7	69	17.3
Moderately differentiated	529	66.4	264	66.3
Poorly differentiated	138	17.3	64	16.1
Undifferentiation	5	0.6	1	0.3
**Surgical approach**				
Ivro-Lewis esophagectomy	580	72.8	282	70.9
McKeownesophagectomy	172	21.6	91	22.9
Left transthoracic esophagectomy	45	5.6	25	6.3
**Number of examined lymph nodes**				
<10	42	5.3	25	6.3
10–19	241	30.2	103	25.9
20–29	286	35.9	168	42.2
30–39	151	18.9	69	17.3
40–49	54	6.8	18	4.5
≥50	23	2.9	15	3.8
Mean, Median (Range)	24.81, 23 (3–69)		24.6, 23 (1–62)	
**Number of positive lymph nodes (N stage)**				
0 (N0)	435	54.6	208	52.3
1–2 (N1)	190	23.8	99	24.9
3–6 (N2)	123	15.4	62	15.6
≥7 (N3)	49	6.1	29	7.3
Mean, Median (Range)	1.68, 0 (0–29)		1.73, 0 (0–26)	
**Extent of number of positive lymph nodes (Cervical, upper mediastinal, lower mediastinal and abdomial fields)**				
0	435	54.6	208		52.3	
1 field	209	26.2	114		28.6	
2 fields	121	15.2	59		14.8	
3 fields	29	3.6	17		4.3	
4 fields	3	0.4	0		0	
**Depth of invasion (T stage)**						
Epithelial lamina	9	1.1	4	1.0
Lamina propria and muscularis mucosae	27	3.4	17	4.3
Submucosa	94	11.8	40	10.1
Superficial and deep muscular layer	143	17.9	64	16.1
Adventitia	480	60.2	238	59.8
Adjacent structure	44	5.5	35	8.8
**Vessel infiltration**				
No	660	82.8	341	85.7
Yes	137	17.2	57	14.3
**TNM staging (6** ^**th**^ **edition)**				
I a	9	1.1	5	1.3
I b	101	12.7	51	12.8
II a	311	39	145	36.40
II b	69	8.7	27	6.8
III	307	38.5	170	42.7
**TNM staging (7** ^**th**^ **edition)**				
I a	23	2.9	15	3.8
I b	118	14.8	52	13.1
II a	155	19.4	62	15.6
II b	177	22.2	92	23.1
III a	156	19.6	82	20.6
III b	93	11.7	45	11.3
III c	75	9.4	60	12.6
**Overall Survival**				
3-year survival rate	46%		44%	
5-year survival rate	30%		35%	
Mean, Median (Range)	30.85, 25.00(1–127)		31.16, 24.50(2–117)	

580 (72.8%) and 282 (70.9%) patients in the training and validation cohorts underwent Ivor Lewis esophagectomy, respectively. A left thoracic approach was performed for 45 patients (5.6%) and 25 patients (6.3%) in the training and validation cohorts, respectively. McKeown esophagectomy was performed respectively for 172 patients (21.6%) and 91 patients (22.9%) in the training and validation cohorts.

The 3-year survival rates were 46% and 44% in the training and validation cohorts, respectively. The 5-year survival rates were 30% and 35% in the training and validation cohorts, respectively. In the training cohort, the median survival time was 30.83 months with a range of 1 to 127 months. In the validation cohort, the median survival time was31.36 months with a range of 2 to 117.

Multivariate analyses were performed to fit all variables in the Cox PH regression model, and variables significantly associated with OS were selected using the backward step-down selection method. The clinicopathological factors were identified as independent risk factors for OS, including tumor length, surgical approach, number of examined lymph node, number of positive lymph node, extent of lymph node, grade, and depth of invasion (Table 2).

**Table 2:** Identified variables by Cox Multivariate Regression Analysis in the training cohort.

Variable	B	*p*	Exp(B)	95.0% CI for Exp(B)
**Tumor length (cm)**				
<3.0			1	
3.0–4.9	0.109	0.592	1.116	0.746–1.669
5.0–7.9	0.365	0.074	1.441	0.963–2.157
≥8	0.590	0.026	1.805	1.072–3.040
**Number of positive lymph nodes (N stage)**				
0 (N0)			1	
1–2 (N1)	0.426	0.019	1.532	1.072–2.189
3–6 (N2)	0.821	0.001	2.274	1.384–3.736
≥7 (N3)	1.299	0.000	3.666	1.923–6.987
**Grade**				
Well-differentiated			1	
Moderately differentiated	0.339	0.041	1.403	1.012–1.945
Poorly differentiated	0.696	0.000	2.007	1.382–2.914
Undifferentiation	1.271	0.017	3.565	1.254–10.134
**Surgical approach** *	0.129	0.124	1.137	0.965–1.341
**Examined of lymph node** *	-0.176	0.000	0.838	0.762–0.921
**Extent of lymph node** *	0.172	0.135	1.187	0.947–1.488
**Depth of invasion** *	0.343	0.000	1.410	1.201–1.654

*not treated as factorial variable, due to the violation the PH assumption in some factorial levels of the variable.

**Fig 1:**
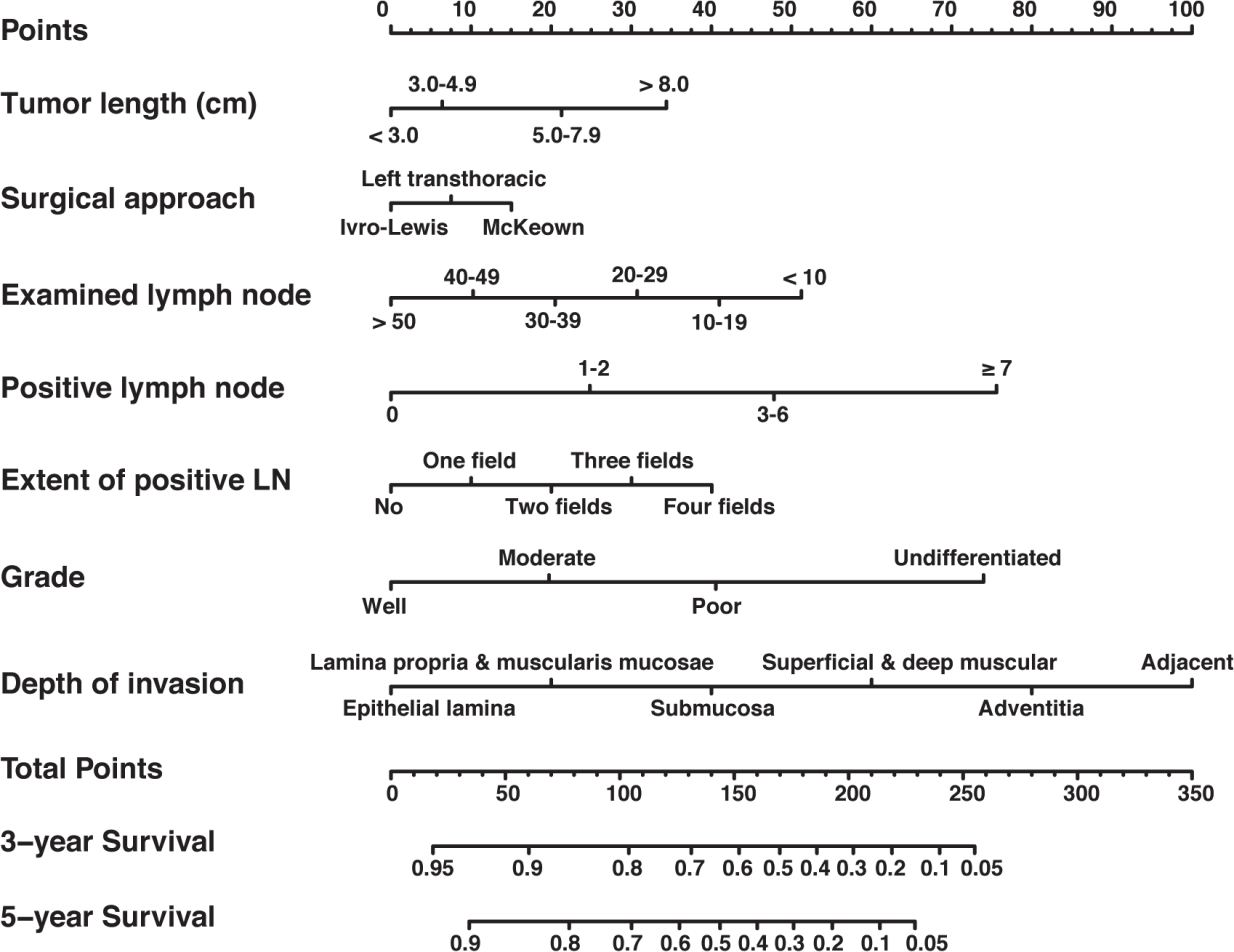
Nomogram for predicting 3-year and 5-year overall survival after radical esophagectomy for esophageal squamous-cell cancer. To calculate the survival rate of each individual patient, points for each of the factors were first identified on the uppermost point-scale, and then the total points from all factors were added up and projected on the bottom point-scale to indicate the probability of 3-year and 5-year survival. Abbreviation: LN, lymph nodes.

**Fig 2:**
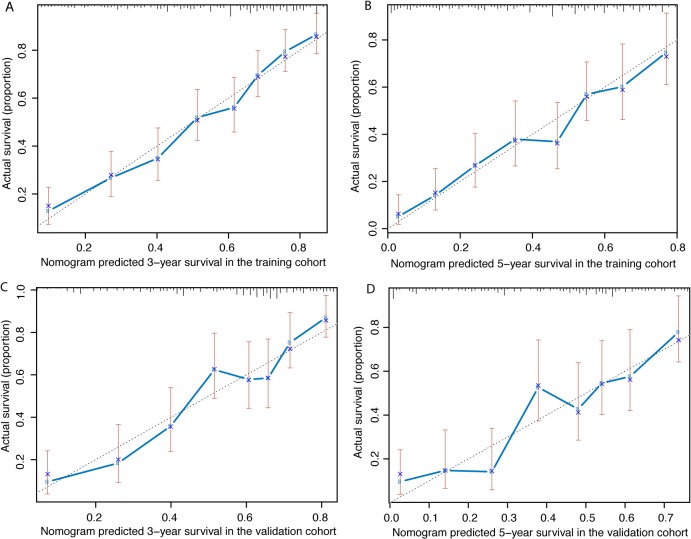
Calibration curve of the nomogram model. The X-axis and Y-axis represent the nomogram-predicted and actual survival probabilities, respectively. (A) Three-year and (B) Five-year overall survival in the training cohort. (C) Three-year and (D) Five-year overall survival in the validation cohort.

Fig 1 shows the prognostic nomogram predicting 3- and 5-year overall survival developed from the results of multivariate analysis based on all significant risk factors using the training cohort. The C-index for predicting overall survival was 0.725 (95% CI, 0.694–0.756) using bootstrap resampling approach. Calibration plot using bootstrap resampling of the training cohort was illustrated in Fig 2A and 2B, showing that the nomogram predicted 3- and 5-year survival probabilities agrees optimally with the actual observation.

The resulting model was externally validated using the bootstrap method in the validation cohort. The bootstrap corrected C-index was 0.715 (95% CI, 0.671–0.759). Calibration plot of the nomogram using the validation cohort was illustrated in Fig 2C and 2D. Calibration curves for 3- and 5-year OS show good agreement between the predicted and observed survival probabilities.

Discriminative ability of the nomogram and that of both the sixth and seventh AJCC TNM classifications were determined using the C-index. In the training cohort, the C-indices of the discrimination of the sixth and seventh editions of the TNM system were 0.664 (95% CI, 0.635–0.693) and 0.696 (95% CI, 0.665–0.727), respectively. Both C-indices were significantly smaller than the C-index (0.725) of the nomogram discrimination (*P*-values < 0.0001 in the sixth TNM edition and *P*-values = 0.0003 in the seventh TNM edition). In the validation cohort, the C-index (0.715) of the nomogram discrimination was also significantly higher than the C-index (0.66, 95% CI = 0.618–0.702) of the sixth TNM classification (*P*-value = 0.0004), and the C-index (0.7, 95% CI = 0.656–0.743) of the seventh TNM classification (*P*-value = 0.0086). The results suggest that our nomogram is better than the AJCC TNM classifications in prognostic prediction.

**Fig 3:**
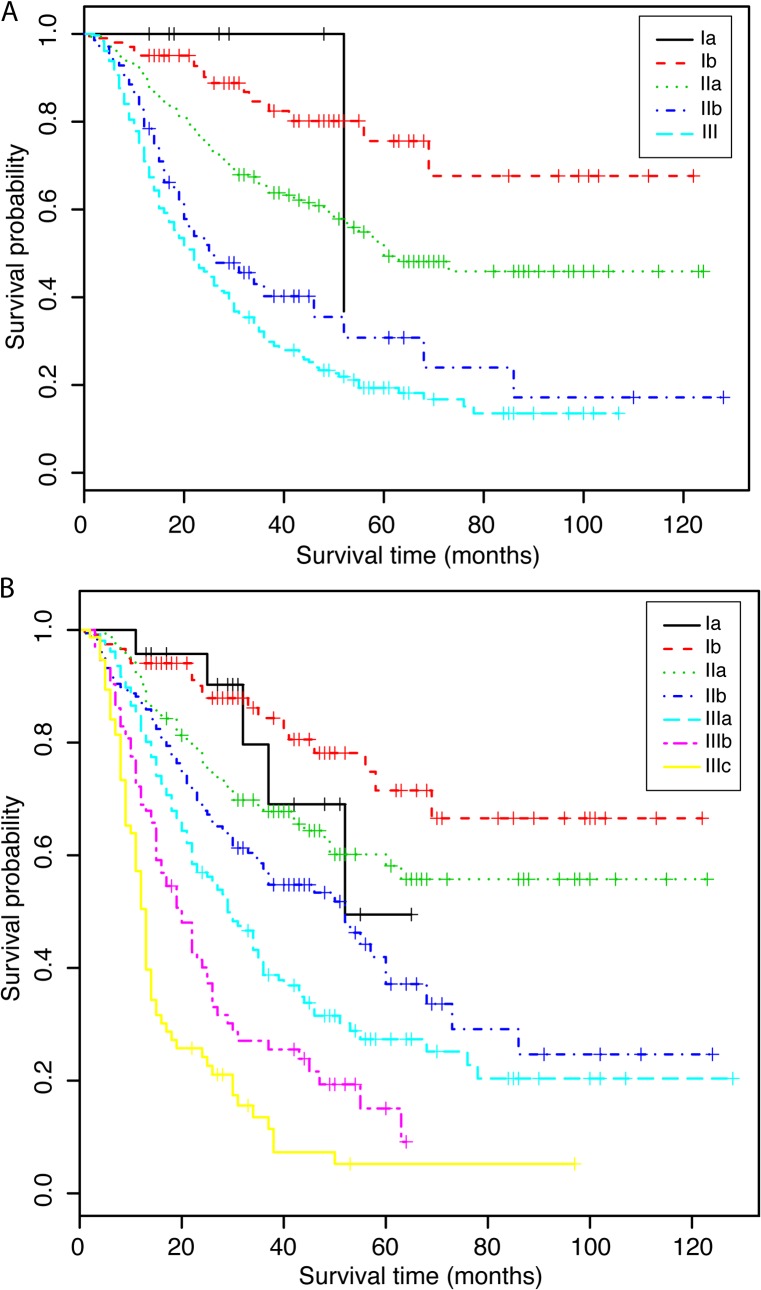
Kaplan-Meier curve of the training cohort stratified for (A) the sixth edition, and (B) the seventh edition of the TNM staging system.

**Fig 4:**
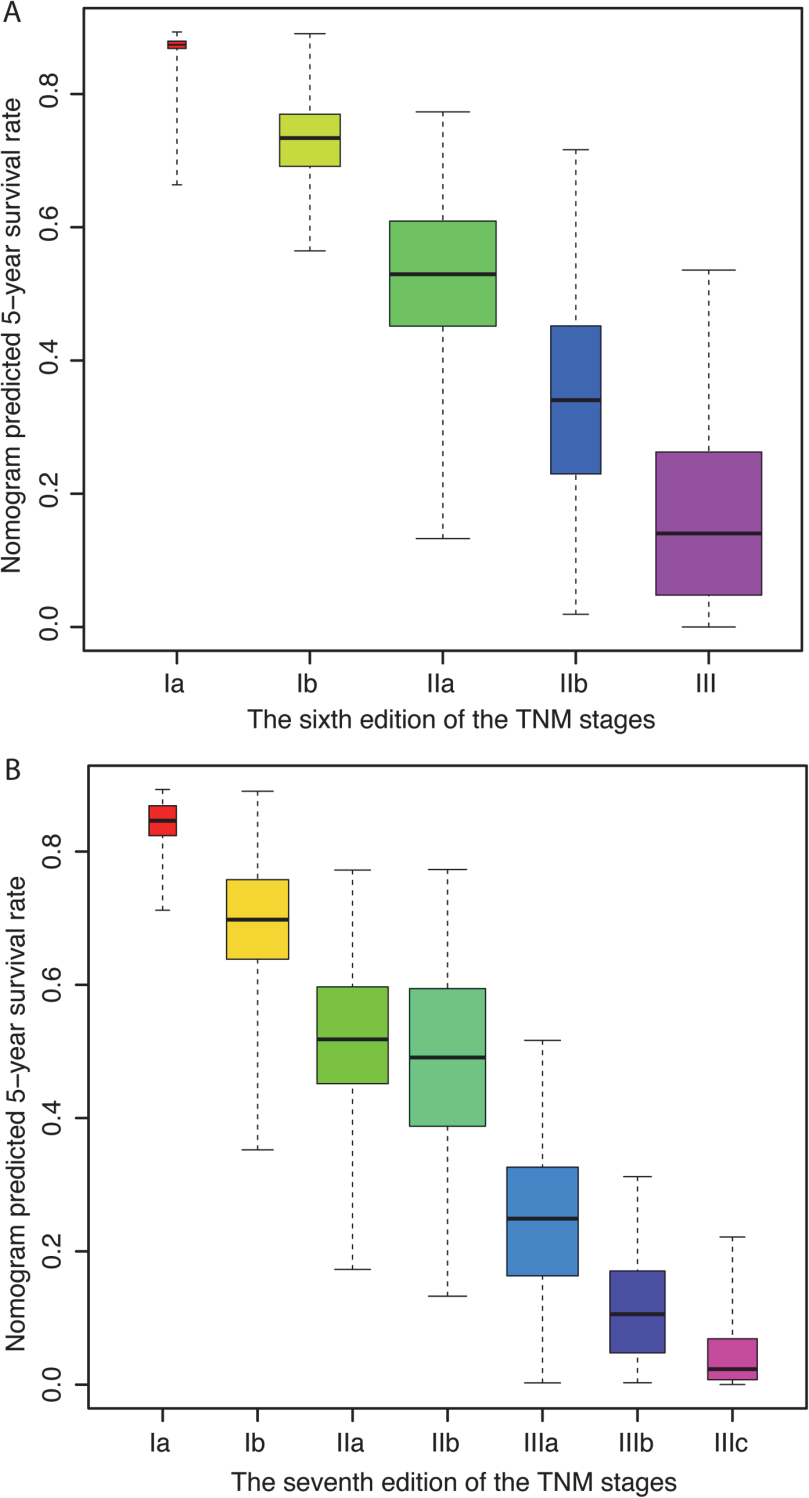
Distribution of the nomogram predicted 5-year survival according to (A) the sixth edition, and (B) the seventh edition of the TNM staging system. The predicted survival probabilities in each of the TNM stages exhibit a wide range of variation.

Fig 3 illustrates that in the training cohort, both the sixth and seventh editions of the AJCC TNM system were able to stratify patients from stage II to all the later stages, but failed to distinguish patients from stage I to stages II and stage III. Fig 4 illustrates the 5-year survival predicted by the nomogram in the training cohort for each stage of the sixth and seventh editions of the AJCC TNM system. The overall survival predicted by the nomogram was distinctive in each of the TNM stages, the higher the stages and the lower the survival of the patients. Within each of the stages, however, the survival rates show a wide range of variations, and overlap with adjacent stages.

## Discussion

ESCC is an aggressive cancer with high incidence and death rate in China. It is generally agreed that comprehensive therapy should be considered for the treatment of ESCC, and neoadjuvant therapy has become an acceptable approach[14–16,29]. However, it remains controversial regarding the question of whether adjuvant therapy can improve the survival rates of ESCC patient after the treatment of R0 resection[17,20,21]. On the basis of the largest available cohort of patients with ESCC treated with radical esophagectomy alone, this study developed the first prognostic nomogram for ESCC based on seven significant clinicopathological factors.

We performed both internal and external validation to assess the predictive accuracy of the nomogram. The discriminative ability of the nomogram was supported by a C-index of 0.725 using internal validation for the training cohort, and of 0.715 using external validation for the validation cohort. Although the predictive accuracy decreased some in the validation cohort, the C-index values suggest a sufficient level of accuracy in both cohorts. Calibration curves using both the training and validation cohorts demonstrated that the predicted survival probabilities were in good agreement with the actual survival rates.

The TNM classification was the most commonly used system for survival prediction for patients with ESCC. However, the predictive accuracy of the TNM staging systems for ESCC was significantly less than that of the nomogram constructed by this study, as indicated by the C-indices. Moreover, we demonstrated that the survival probabilities of patients within each of the TNM stages varied diversely, and overlap somewhat with adjacent stages. This situation also makes the TNM staging system difficult for individualized estimates of survival in practice.

Our prediction model confirmed that the TNM factors, including depth of tumor invasion (T), number of positive lymph nodes (N) and grade, were independent prognostic predictors. However, tumor location, which was integrated into the seventh edition of TNM classification, was not an independent prognostic predictor for ESCC in this study. This finding is consistent with that of some recent studies, which have also excluded tumor location as a covariate significantly associated with survival for ESCC in Chinese population[43,44]. In addition, this study identified four new significant risk factors for ESCC, including tumor length, surgical approach, number of examined lymph nodes, and extent of positive lymph nodes. The predictive accuracy of the nomogram was increased significantly with the inclusion of those non-TNM factors.

Surgery is the major treatment method for patients with ESCC. Transthoracic esophagectomy is the most widely used surgery for esophageal cancer worldwide[45]. The surgical procedure can be performed through a right or left thoracotomy incision depending on the experience of the surgeon and tumor location. In China, the mainstay surgical approaches for patients with ESCC are Ivor Lewis esophagectomy (right thoracotomy and Laparotomy), McKeown esophagectomy (right thoracotomy and Laparotomy and cervical anastomosis), and left transthoracic esophagectomy. Studies have reported that 3-year survival rates of patients who underwent esophagectomy through right incision were higher than those through left incision[46,47]. Our study demonstrates that patients treated by Ivor Lewis esophagectomy exhibited better survival than those treated by the other two approaches (Fig 1). Compared to the other two surgical approaches, Ivor Lewis esophagectomy has the advantages to provide adequate exposure to ensure a thorough resection of tumor and a sufficient radial margin, and allow a wider extent of lymphadenectomy with limited postoperative complications. We think that these surgical advantages of Ivor Lewis may contribute to the improvement of patient outcome by decreasing the risk of recurrence. The identification of surgical approach as an independent predictor suggests that Ivor Lewis esophagectomy should be considered as the primary treatment for patients with ESCC, especially for tumor occurred in the middle or lower esophagus.

This study demonstrates that increasing the numbers of examined lymph nodes is able to enhance the survival of patients. The average number of examined lymph nodes is about 25 in our study. This result is consistent with that of several recent studies, which also identified number of examined lymph nodes as an independent predictor for ESCC, and suggested a minimum removal of 23 lymph nodes to maximize patient outcome[48,49]. In addition, we found that extent of positive lymph node was a covariate associated with survival for ESCC. Usually, extent of positive lymph node reflects the scope of metastasis, which is divided as cervical, upper mediastinal, lower mediastinal and abodomial fields. Studies have shown that extent of lymph node involvement in ESCC plays an important role in prognostic prediction, and 5-year survival rate of ESCC patients with positive lymph node in one-field was 50%, two-fields was 29% and three-field was 11%[50,51]. Consistent with these previous studies, our nomogram model also demonstrated that patients with multiple fields of lymph node involvement show worse survival than those with none or one field of lymph node involvement.

Tumor length was not adopted as a covariate for ESCC by the seventh edition of AJCC TNM staging system. In contrast, some studies have suggested that tumor length, which represents longitudinal spreading of the cancerous cells, can affect the overall survival of patients with ESCC[17,18]. Similarly, this study identified that tumor length is negatively correlated with patient survival. As indicated in our nomogram, patients with a tumor length ≥3cm exhibit higher death risk than those with a tumor length <3cm.

The present study has several limitations. First, this study only has samples from a single institution to establish the nomogram. Second, our data set only contains a small number of patients treated with left transthoracic esophagectomy: 6.4% (n = 69) in the training cohort and 5.8% (n = 29) in the validation cohort. A more representative sample of patients treated with left transthoracic esophagectomy will be needed to further assess the survival benefits of this surgical technique in future studies.

## Conclusions

In summary, we developed a nomogram for predicting 3-year and 5-year overall survival for patients with ESCC after radical esophagectomy. The predictive capability of the nomogram was validated internally and externally using the training and validation cohorts, respectively. The nomogram demonstrates its superior performance than the TNM staging system in prognostic prediction, and has the potential for individualized estimates of survival in daily clinical practice.
